# Oxidative stress markers are associated to vascular recurrence in non-cardioembolic stroke patients non-treated with statins

**DOI:** 10.1186/1471-2377-12-65

**Published:** 2012-08-03

**Authors:** David Brea, Jaume Roquer, Joaquín Serena, Tomás Segura, José Castillo

**Affiliations:** 1 Clinical Neuroscience Research Laboratory, Department of Neurology, Hospital Clínico Universitario, University of Santiago de Compostela, Santiago de Compostela, Spain; 2 Department of Neurology, Hospital Universitari del Mar, Parc de Salut Mar, Barcelona, Spain; 3 Department of Neurology, Hospital Universitario Dr. Josep Trueta, Institut d’Investigació Biomèdica de Girona, Girona, Spain; 4 Servicio de Neurología, Hospital Universitario de Albacete, Albacete, Spain

**Keywords:** Ischemic stroke, Vascular recurrence, Atherothrombosis, Oxidative stress

## Abstract

**Background:**

Since atherogenesis is related to oxidative stress, our objective was to study the association of oxidative stress markers with the vascular recurrence in non-cardioembolic stroke.

**Methods:**

Atherosclerotic and oxidative stress markers were evaluated on admission, in 477 patients suffering from a first non-cardioembolic stroke. Patients were followed at 6 and 12 months after inclusion, recording cardiovascular events. As markers of endothelial oxidative stress we used oxidized LDL, Cu/Zn superoxide dismutase and 8-OH deoxiguanosine. 136 patients were being treated with statins at the moment of serum samples acquisition.

**Results:**

Patients who suffered vascular recurrence or vascular-origin death had higher levels of 8-OHDG (40.06±24.70vs33.11±15.18;p=0.003). We also found associations between vascular recurrence or vascular origin death and Cu/ZnSOD (OR,1.02; 95%CI,1.00-1.03;p=0.0001) and 8-OHDG (OR,1.12;95%CI,1.08-1.16;p<0.0001) in a subgroup of 333 patients that were not in treatment with statins on admission. We also found associations between 8-OHDG and intima media thickness (IMT) (OR,1.13;95%CI,1.09-1.16;p<0.0001), presence of ipsilatieral stenosis≥50% (OR,1.03;95%CI1.00-1.05;p=0.007) and other atherosclerotic plaque characteristics.

**Conclusions:**

Specific oxidative stress markers were found to be markers of atherosclerosis plaque types and vascular recurrence in non-statins treated patients at admission.

## Background

Atherosclerosis and atherothrombosis involve inflammation, oxidative stress, plaque disruption, platelet activation and aggregation, and thrombus formation. Thrombosis superimposed on progressive narrowing of atherosclerotic arteries may result in sudden arterial occlusion or in microembolization leading to acute coronary syndrome, ischemic stroke, transient ischemic attack, or other symptoms [1,2].

Atherothrombosis is associated with significant morbidity and mortality, both from the initial and subsequent secondary vascular events, especially within the first year [3]. It has previously been shown that, the risk of vascular recurrence within the first year after a first stroke is 15 times higher than the risk in the general population [4]. In addition, if we only consider non-cardioembolic stroke patients, 20-40% of patients will suffer recurrence within five years after the first stroke event [4]. However, there are no markers that allow the identification of patients at high risk of vascular recurrence.

Atherogenesis is associated with oxidative stress and reactive oxygen species. The generation of reactive oxygen species (ROS) is important in both normal physiology and in the pathogenesis of many diseases. Accumulation of ROS may be accompanied by the production of reactive nitrogen species. Under physiological conditions, cells defend themselves against ROS damage through antioxidants that remove free radical intermediates and inhibit oxidation. An imbalance between endogenous oxidants and antioxidants results in oxidative stress, that contributes to vascular dysfunction and atherogenesis [5].

Oxidative stress plays a key role at several steps of atherogenesis. Thus, for example LDL is oxidatively modified by endothelial cells, vascular smooth muscle cells, and monocytes. Macrophages within the vessel wall internalize ox-LDL via scavenger receptors, and develop into lipid-rich “foam cells”. Evidence that LDL oxidation occurs in vivo is supported by the reaction of ox-LDL antibodies with atherosclerotic lesions [6]. Other enzymes or markers of oxidative stress are also important in atherogenesis process. In addition to ox-LDL, enzymes that participates in oxidative stress regulation, for example superoxide dismutase (SOD), are increased in atherosclerotic lesions and colocalized with lipid-laden macrophages in atherosclerotic vessels of apo(E)-deficient mice [7].

Since oxidative stress participates in the atherogenesis process and this process is an important cause of recurrence in non-cardioembolic stroke, our aim was to analyze the association of several markers of oxidative stress with the recurrence of vascular events and with atherosclerotic plaque characteristics.

## Results

### Population characteristics

Four hundred seventy seven independent patients older than 60 years suffering from a first non-cardioembolic stroke were included in the study (76.9% of total patients). The mean age of these patients was 71.8 ± 7.5 years; 331 of them were men (69.39%). Distribution by stroke subtype was 61.43% atherothrombotic (n = 293), 19.70% lacunar (n = 94) and 18.87% were cryptogenic (n = 90) (Table 1). Levels of oxidative stress markers at admission on this group of patients were; 54.72 [45.53–68.74] ng/mL for oxLDL, 46.12 [31.56-71.21] ng/mL for Cu/Zn SOD, and 34.10 ± 17.01 ng/mL for 8-OHDG. 136 patients (38.5%) were in treatment with statins at admission. These patients had greater percentage of vascular risk factors and lower levels of 8-OHDG.

**Table 1:** Baseline clinical characteristics, vascular risk factors, stroke subtype, biochemical parameters and neuroimaging findings in patients with and without recurrence

	**Primary end-point**		
	**No n = 409**	**Yes n = 68**	**p**
Age, years	70.9 ± 7.2	71.4 ± 8.2	0.687
Male, %	68.7	73.5	0.479
Weight, kg	75.1 ± 11.8	78.5 ± 14.5	0.106
Waist circumference, cm	101.3 ± 12.6	103.7 ± 14.4	0.203
Systolic blood pressure, mm Hg	152.8 ± 25.2	161.1 ± 26.4	0.017
Diastolic blood pressure, mm Hg	82.9 ± 13.0	85.2 ± 14.3	0.141
Arterial hypertension, %	72.5	69.1	0.562
Diabetes mellitus, %	34.4	45.6	0.078
Active smoker, %	29.6	26.5	0.667
Alcohol intake, %	4.9	8.8	0.242
Dyslipemia, %	44.2	48.5	0.513
Ischaemic CHD, %	11.5	16.2	0.316
Symptomatic PAD, %	6.0	23.5	<0.0001
Treatment before index stroke			
Antiplatelets, %	25.2	28.4	0.651
Statins, %	28.6	31.3	0.664
Antihypertensive, %	62.2	60.3	0.788
Hypoglycemics, %	26.9	36.4	0.139
Treatment at discharge			
Antiplatelets, %	99.3	100	0.628
Statins, %	73.8	73.5	0.533
Antihypertensive, %	76.8	76.1	0.505
Hypoglycemics, %	33.0	35.3	0.404
Stroke subtype			<0.0001
Atherothrombotic probable, %	14.8	35.3	
Atherothrombotic posible, %	43.7	41.2	
Lacunar, %	21.0	13.2	
Cryptogenic, %	20.5	10.3	
Modified Rankin at discharge	1 [0, 2]	2 [1, 2]	0.034
ABI	1.00 ± 0.23	0.92 ± 0.29	<0.0001
IMT, mm	0.91 ± 0.34	0.96 ± 0.44	0.043
ICA stenosis > 50 % homolateral, %	14.8	35.8	<0.0001
ICA stenosis > 50 % contralateral, %	5.7	16.4	0.004
Echogenicity of plaques			0.493
Type I, %	15.0	10.7	
Type II, %	14.7	17.9	
Type III, %	24.5	21.4	
Type IV, %	37.9	46.4	
Type V, %	8.2	3.6	
Plaques surface			0.001
Smooth and even, %	75.0	50.0	
Uneven, %	23.7	48.3	
Ulcerated, %	1.3	1.7	
8-OHDG (ng/mL)	33.11 ± 15.18	40.06 ± 24.70	0.003
Cu/Zn SOD (ng/mL)	44.37[31.06-70.06]	52.75 [36.73-89.59]	0.062
ox-LDL (ng/mL)	54.83 [45.71-69.36]	53.43[43.34-62.52]	0.241

The study was approved by the ethical committee of each centre and informed consent was obtained from patients or relatives.

### Primary endpoint; vascular recurrence or vascular origin death and oxidative stress markers

Univariate analysis revealed association between systolic blood pressure (p = 0.017) symptomatic PAD (p < 0.0001), stroke subtype (p < 0.0001), mRS on discharged (p = 0.034), ABI (p < 0.0001), IMT (p = 0.043), ipsilateral stenosis in ICA (p < 0.0001), contralateral stenosis in ICA (p = 0.004), and plaque surface (P = 0.001) and higher risk of vascular events on follow-up. In addition, patients who suffered vascular recurrence or vascular death had higher levels of 8-OHDG (40.06 ± 24.70 vs 33.11 ± 15.18; p = 0.003). However, no differences were found in ox-LDL on admission (53.43 [43.34-62.52] vs 54.83 [45.71-69.36]; p = 0.241) or Cu/Zn SOD on admission (52.75 [36.73-89.59] vs 44.37 [31.06-70.06]; p = 0.062). Logistic regression analysis including all significant variables in the univariate analysis revealed no independent association between 8-OHDG levels and vascular recurrence or vascular death(OR, 1.02; 95%CI, 0.94-1.04; p = 0.197).

When the primary endpoint was categorized in vascular-origin death, non-vascular origin death, vascular recurrence and non-events, association was found with Cu/ZnSOD (65.84 [33.80-111.99], 52.59 [27.94-63.40], 52.18 [37.09-96.43], 44.37 [31.06-70.06] respectively; p = 0.027 for all comparison) and with 8-OHDG (32.04 ± 25.17; 36.69 ± 18.03; 41.46 ± 25.20; 33.11 ± 15.18 respectively; p = 0.009 for all comparison), but not with ox-LDL (p = 0.706).

### Secondary endpoint; atherosclerotic markers and oxidative stress markers

Since oxidative stress markers could be associated with atherogenesis and atherosclerotic plaque progression, we analyzed the association between ox-LDL, Cu/Zn SOD and 8-OHDG and atherosclerotic markers, such as ABI, IMT, stenosis grade and plaque echogenicity. No association was found between ABI and oxidative stress markers that were analyzed (ox-LDL, Cu/Zn SOD or 8-OHDG). Association was found between IMT and Cu/ZnSOD (Spearman coefficient 0.099, p = 0.034) and between IMT and 8-OHDG (Pearson coefficient 0.133; P = 0.004). When IMT was categorized in IMT < 1.1 mm or IMT ≥ 1.1 mm, association was only found for 8-OHDG (32.60 ± 14.92 vs 38.51 ± 21.55, respectively; p = 0.001). This association persisted in logistic regression analysis (OR, 1.02; 95% CI, 1.00-1.03; p = 0.004). No association was found between oxidative stress markers and carotid plaques characteristics, such as stenosis or plaques characteristics (smooth, irregular, ulcerated plaques) (data not shown).

### Vascular recurrence and oxidative stress markers in a subgroup of patients

In a post-hoc analysis we observed that 144 patients from 477 in which serum sample was available were being treated with statins at the moment of serum sample acquisition. Since statins have pleiotropic effects including antioxidant properties, we decided to re-analyze the potential value of oxidative stress markers in the subgroup of patients that were not treated with statins at the moment of serum extraction.

Three-hundred and thirty three patients were not being treated with statins when serum sample was extracted (mean age 71.29 ± 7.43). Two hundred thirty seven patients were men (71.17%), 193 were atherothrombotic patients (57.95%), 72 were lacunar (21.62%) and 68 were cryptogenic (21.72%). Levels of oxidative stress markers at admission on this subgroup of patients were; 54.92 [45.30-68.41] ng/mL for oxLDL, 47.45 [32.82-70.78] ng/mL for Cu/Zn SOD, and 39.85 ± 13.97 ng/mL for 8-OHDG.

**Figure 1:**
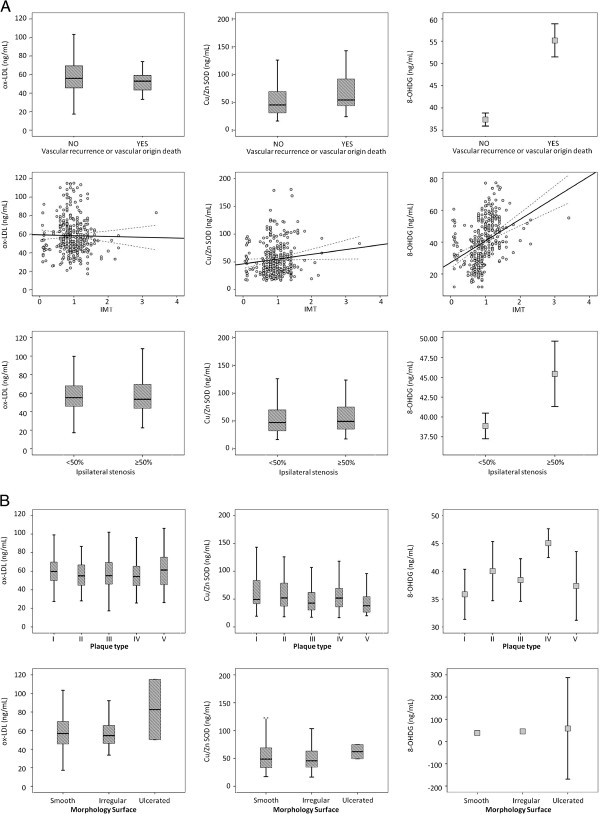
Oxidative stress markers (ox-LDL, Cu/Zn SOD and 8-OHDG) values classified according to: (1A)the primary endpoint (vascular recurrence) or secondary endpoints (atherosclerotic characteristics); (1B) according to the plaque type or the plaque surface.

In this subgroup of patients, patients who suffered vascular recurrence or vascular death showed higher levels of Cu/Zn SOD (45.39 [30.99-69.31] vs 54.30[43.60-92.86]; p = 0.001)) and higher levels of 8-OHDG (37.40 ± 12.57 vs 55.17 ± 12.56; p < 0.0001) (Figure 1A). When logistic regression analysis were performed including all the significant variables in the univariate analysis together with oxidative stress markers, association between vascular recurrence or vascular origin death and Cu/ZnSOD (OR, 1.02; 95% CI, 1.00-1.03; p = 0.0001) and 8-OHDG (OR, 1.12; 95% CI, 1.08-1.16; p < 0.0001) persisted.

We also analyzed, in this subgroup of patients, the relationship between oxidative stress markers and atherosclerosis markers. We did not find any association between oxidative stress markers and ABI, however association was found between IMT and 8-OHDG (Pearson coefficient 0.502, p < 0.0001) (Figure 1A). In addition, when IMT was categorized in IMT < 1.1 mm or IMT ≥ 1.1 mm, Cu/Sn SOD (52.57 ± 28.43 vs. 60.45 ± 32.63, p = 0.033) and 8OHDG (36.24 ± 12.92 vs 50.05 ± 11.71 p < 0.0001) were significantly higher in patients with IMT ≥ 1.1. After adjusting by the significant variables in the univariate study, association between IMT ≥ 1.1 and 8-OHDG persisted in logistic regression analysis (OR, 1.09; 95% CI, 1.06-1.12; p < 0.0001).

Analysis of relationship between oxidative stress markers and atherosclerotic plaques characteristics, also showed some positive results. When we analyzed oxidative stress markers in relation to stenosis grade of atherosclerotic plaques, we found that 8-OHDG levels were higher in patients with stenosis ≥50% in the ipsilateral carotid (45.42 ± 15.07 vs 38.86 ± 13.55, p = 0.005) (Figure 1A). In addition we also found that 8-OHDG was higher in patients with stenosis in the contralateral carotid (46.36 ± 13.00 vs 39.65 ± 13.90, p = 0.034). However, when adjusted models were performed, 8-OHDG only remained associated with the presence of ipsilateral stenosis ≥50% (OR, 1.03; 95 % CI 1.00-1.05; p = 0.007).

Analyzing plaques types, we observed that 38 patients showed anechoic plaques (type I), 34 patients showed hypoechoic plaques (type II), 61 patients showed echoic plaques (type III), 96 patients showed hyperechoic plaques (type IV) and 21 patients showed non-classified plaques (type V). In these groups of patients, 8-OHDG levels were different with p = 0.002 (35.84 ± 13.66, 40.00 ± 15.21, 38.41 ± 14.92, 45.02 ± 12.76, 37.35 ± 13.54, respectively) (Figure 1B).

Finally, atherosclerotic plaques were classified according to morphology surface in smooth (n = 190), irregular (n = 68) or ulcerated (n = 2). Analysis of oxidative stress markers showed significant differences in 8-OHDG levels with p < 0.0001 in these patients (38.53 ± 13.52; 45.87 ± 14.53; 59.12 ± 25.40 respectively), although the small number of patients with ulcerated plaques makes these results suspect (Figure 1B). If plaque surface is categorized in regular (smooth) and irregular (irregular plus ulcerated), multivariate analysis does not show independent significance for 8-OHDG (OR, 1.14; 95% CI 0.99-1.31; p = 0.069).

## Discussion

In this study we have shown that oxidative stress markers levels correlate with risk of recurrent stroke and cardiac death in patients not receiving statins at the time of presentation. We also demonstrated association between oxidative stress markers and atherosclerotic plaque characteristics in this subgroup of patients.

Patients with atherothrombosis have been found to have relatively high rates of cardiovascular events [8-15]. Given that atherosclerosis is a systemic condition, the vascular event can occur in the same vascular territory or, as is frequently the case in stroke patients, in a different one. Although stroke recurrence in non-cardioembolic patients is important, no markers are available to identify patients with high risk of vascular recurrence or vascular-origin death. In this study we have analyzed three oxidative stress markers (ox-LDL, Cu/Zn SOD and 8OHDG) as potential markers of risk for recurrent stroke or vascular death. We also evaluate the relationship between these markers and other classic markers of risk (IMT, carotid stenosis grade and plaque echogenicity and surface characteristics).

First, it is important to note that we found no association between ox-LDL, Cu/Zn SOD or 8-OHDG and recurrent stroke in our study group. However when data was re-analyzed, excluding patients receiving statins, we found an association between Cu/Zn SOD and 8-OHDG levels and recurrent stroke and vascular death. In addition, we found associations between 8-OHDG and IMT and carotid stenosis grade. We also found differences in 8-OHDG in relation to atherosclerotic plaque surface.

The fact that we have found associations between oxidative stress biomarkers and atherosclerosis and vascular recurrence in patients with non-cardioembolic stroke without statin treatment but not in the general non-cardioembolic group of patients, indicates that statin treatment affects oxidative stress marker levels.

Statins are a group of lipid-lowering drugs, 3-hydroxy-3-methylglutaryl-coenzyme A (HMG-CoA) reductase inhibitors, used in the prevention and treatment of cardiovascular diseases. Statins also possess cholesterol-independent or “pleiotropic” effects which include improvement of endothelial function, stabilization of atherosclerotic plaques, inhibition of oxidative stress and inflammation, and a reduction of thrombogenic response [16]. These beneficial effects of statins are, at least in part, mediated by an effect on eNOS (endothelial Nitric Oxide Synthase) [17-19]. But statins also ameliorate oxidative stress [20] by reducing the expression and/or activity of NADPH oxidase [21]. These effects may be partly responsible for the antiatherogenic action of statins [22-24]. Since statins have effects on eNOS and NADPH oxidase, it is not surprising that statins treatment could influence levels of oxidative stress markers, such as 8-OHDG or Cu/Zn SOD. Further prospective studies could evaluate the potential of 8-OHDG or Cu/Zn SOD as prognostic markers.

Our data suggest that oxidative stress marker levels correlate with outcomes only in patients not treated with statins. One possible explanation of this is that statins reduce oxidative stress.

## Conclusions

Specific oxidative stress markers were found to be markers of atherosclerosis plaque types and vascular recurrence. Thus, 8-OHDG and Cu/Zn SOD could be useful markers to identify patients with high risk of vascular recurrence or vascular death and 8-OHDG could be useful to identify particular atherosclerotic plaques characteristics, such as echogenicity, stenosis grade or plaque surface characteristics in patients who are not receiving statin therapy.

## Methods

### Study design

From January to July 2009, 42 neurology departments in Spain performed the ARTICO study. This study included patients with non-cardioembolic stroke with age ≥60 years. All patients were admitted to neurology departments and evaluated by a neurologist before inclusion in the study. All patients were treated according to the same protocol. The protocol was designed and performed according to the principles of the Helsinki Declaration and was approved by the Ethical Committee of all the participant hospitals (see list of Artico investigators in Appendix). Medical history including vascular risk factors and prior treatment for vascular diseases were recorded. Blood studies including coagulation parameters, brain computed tomography or magnetic resonance, 12-lead ECG, chest radiography, and carotid ultrasonography were performed at admission. All patients were treated according to the ARTICO study protocol [25].

Blood samples, taken on admission, were centrifuged at 3000 g for 15 minutes, and immediately frozen and stored at −80°C without any additives.

As markers of endothelial oxidative stress, three markers were analyzed by ELISA kits according to manufacturer’s instructions; one marker of lipid peroxidation, oxidized LDL (ox-LDL; from Mercodia); one enzymatic marker, Cu/Zn superoxide dismutase (Cu/Zn SOD, from Bender Medsystems), and one nucleic acid lesion marker, 8-OH-2deoxiguanosine (8-OHDG).

As markers of atherosclerotic disease, duplex study of the supraaortic trunk including intima-media thickness (IMT) measurement; quantification of internal carotid stenosis; number, morphology and surface characteristics of carotid plaques; and ankle brachial index (ABI) were performed at study inclusion. Patients were followed at 6 and 12 months after inclusion. Cardiovascular events, including vascular recurrence, ischemic heart disease, symptomatic peripheral arterial disease (PAD), vascular surgery and death of vascular or non-vascular origin were recorded. The modified Rankin scale (mRS) was evaluated at discharge and at 6 and 12 month visits.

Patients were followed at 6 and 12 months after inclusion. Cardiovascular events, including vascular recurrence, ischaemic heart disease, symptomatic peripheral arterial disease (PAD), vascular surgery and death of vascular or non-vascular origin were recorded. The modified Rankin scale (mRs) was evaluated at discharge and at 6 and 12 month visits. Additional investigations were left at the discretion of the investigator in charge of the patient.

The primary objective of this study was to evaluate the prognostic value of oxidative stress markers to predict death or vascular recurrence in patients that have suffered an episode of ischemic stroke. As secondary objective, relationship between IMT measurement; quantification of internal carotid stenosis; number, morphology and surface characteristics of carotid plaques; ABI, and oxidative stress markers were analyzed.

### Atherothrombotic marker protocol

ABI measurement was performed using an 8 MHz probe and the same Doppler model (ES-100X) in all centers after a consensus meeting. Doppler systolic blood pressure at right and left side in both brachial arteries as well as both the dorsalis pedis artery and posterior tibial arteries were recorded with patients at rest for at least 5 minutes in supine position. The ABI was calculated centrally. Asymptomatic PAD was considered if ABI ≤0.9 and symptomatic PAD when a history of walking impairment, intermittent claudication, ischemic rest pain, and/or nonhealing wounds was present.

Colour-duplex study of the supraaortic trunks was performed on all patients. Carotid or intracranial artery stenosis was quantified using established haemodynamic criteria by echo-Doppler ultrasonography [26]. Each neurosonology department used either their own standardised values or those validated in the literature. Plaques were classified by echogenicity and surface morphology in accordance with consensus criteria [27]. IMT was measured in a still image during diastole in both common carotid arteries (CCA) at the far wall and at least 1 cm below the bifurcation on a 1 cm plaque-free segment. The highest of 6 CCA measurements was taken as the final IMT. To differentiate plaques from just increased IMT, a plaque was defined when focal thickening was ≥1.5 mm. IMT was categorized in IMT < 1.1 mm and IMT ≥ 1.1 mm.

The study was approved by the ethical committee of each centre and informed consent was obtained from patients or relatives.

### Statistical analysis

Results are expressed as percentage, for categorical variables, and as mean (SD) or median [quartiles] for continuous variables, depending on whether they were normally distributed or not. Proportions between groups were compared using the chi-square test. To compare continuous variables between two groups, a Student *t*-test was used for variables normally distributed and a Mann–Whitney test for not normally distributed variables. To assess the statistical significance of more than two continuous variables ANOVA test was used.

Multivariate analyses were performed to evaluate the prognostic value of oxidative stress makers in death or vascular recurrence. Regression analyses were performed after adjusting for the main baseline variables in the univariate analyses (enter approach and probability of entry p <0.05). Results were expressed as adjusted odds ratios (OR) with the corresponding 95% confidence intervals (95% CI). The statistical analysis was performed using SPSS software v16.0.

## Appendix

### List of ARTICO Registry Investigators

Enrique Jiménez Caballero (Hospital Virgen de la Salud, Toledo); Jaume Roquer González (Hospital del Mar, Barcelona); Mercedes Romera (Hospital de Valme, Sevilla); María Jiménez (Hospital Universitario Dr. Josep Trueta, Girona); Jorge García García (Hospital General Universitario, Albacete); Miguel Blanco (Hospital Clínico Universitario, Santiago de Compostela); Vicente Medrano Martínez (Hospital General de Elda, Alicante); Jose M. Ramírez (Hospital San Pedro Alcántara, Cáceres); Exuperio Díez Tejedor (Hospital Universitario La Paz, Madrid); Sergio Calleja (Hospital Central de Asturias, Oviedo); Adriá Arboix Damunt (Hospital Sagrat Cor, Barcelona); Luis García-Tuñon Villaluenga (Hospital de León); Jose A. Egido Herrero (Hospital Clínico Universitario San Carlos, Madrid); Covadonga Fernández Maiztegui (Hospital de Cruces, Barakaldo); Jaime Masjuán Vallejo (Hospital Ramón y Cajal, Madrid); Rosa M. Sánchez Pérez (Hospital Marina Baixa, Alicante); José Miguel Pons Amate (Hospital General Universitario, Valencia); Raúl Espinoso (Hospital Puerta del Mar, Cádiz); Ángel Fernández Díaz (Hospital Comarcal del Bierzo, León); Ernest Palomeras Soler (Hospital de Mataró, Barcelona); Victoria Mejias (Hospital Torrecárdenas, Almería); Carmen Jiménez Martínez (Hospital Universitario Son Dureta, Palma de Mallorca); Manuel Marquez Martínez (Hospital Cliníco Universitario Virgen de la Victoria, Málaga); Alejandro García Escrivá (Hospital de Levante, Alicante); Pere Comas (Hospital de Sant Joan de Deu de Martorell, Barcelona); José Tembl Ferrairo (Hospital Universitario La Fe, Valencia); Rosario Gil (Hospital Clínico Universitario, Valencia); Mayte Martínez (Complejo Hospitalario Donostia); Roberto Belvis (USP Institut Universitari Dexeus, Barcelona); Francisco Moniche Álvarez (Hospital Virgen del Rocío, Sevilla); Javier Abella (Hospital Arquitecto Marcide, La Coruña); Gemma Reig Rosello (Hospital Universitario de La Princesa, Madrid); Oscar Fernández Fernández (Hospital Carlos Haya, Málaga); Isabel Campello (Hospital Royo Villanova, Zaragoza); Toni Figuerola (Hospital de Son Llatzer, Palma de Mallorca); Jordi Sanahuja Montesinos (Hospital Universitari Arnau de Vilanova, Lleida); Enrique Botia Paniagua (Complejo Hospitalario La Mancha Centro, Ciudad Real); Jose Manuel Moltó Jordá (Hospital Francesc de Borja, Valencia); José Luis Martí Vilalta (Hospital de la Santa Creu i Sant Pau, Barcelona); Jose M. Ramírez (Hospital Universitario Infanta Cristina, Badajoz); Elena Vila Herrero (Clínica Santa Elena, Málaga); Marta Ferrero Ros (Hospital General, Segovia).

## Abbreviations

ox-LDL, Oxidized Low Density Lipoprotein; Cu/Zn SOD, Cu Zn Superoxide Dismutase; 8-OHDG, 8-hydroxy-2'-deoxyguanosine; OR, Odds Ratio; IMT, Intima Media Thickness; ROS, Reactive Oxygen Species; LDL, Low Density Lipoprotein; PAD, Peripheral Arterial Disease; mRS, Modified Rankin Scale; ABI, Ankle Brachial Index; ICA, Internal Carotid Artery; HMG-CoA, 3-hydroxy-3-methylglutaryl-coenzyme A; eNOS, Endothelial Nitric Oxide Synthase; NADPH, Nicotinamide Adenine Dinucleotide Phosphate; ECG, Electrocardiogram; CCA, Common Carotid Arteries; SD, Standard Deviation.

## Competing interests

Dr. Roquer, Dr. Serena, Dr. Segura and Dr. Castillo report receiving consulting and advisory board fees from Bristol-Myers Squibb, however the company had nothing to do in the interpretation of data, analysis of results or in the decision to publish.

## Authors’ contributions

All authors have made substantial contributions to conception and design, or acquisition of data, or analysis and interpretation of data; have been involved in drafting the manuscript or revising it critically for important intellectual content; and have given final approval of the version to be published.

## Pre-publication history

The pre-publication history for this paper can be accessed here:

http://www.biomedcentral.com/1471-2377/12/65/prepub
